# Transient disappearance of CD19^+^/CD5^+^ B‐lymphocyte clone in peripheral blood in a patient with CLL during SARS‐CoV‐2‐related mild disease

**DOI:** 10.1002/ccr3.4238

**Published:** 2021-06-22

**Authors:** Remo Barnabei, Giulio Di Michele, Antonio Cellini, Gianfranco Amicosante, Mariagrazia Perilli, Pierangelo Bellio, Alessandra Piccirilli, Giuseppe Celenza

**Affiliations:** ^1^ Clinical Laboratory Regional Hospital "San Salvatore" L'Aquila Italy; ^2^ Department of Infectious Diseases and AIDS Regional Hospital "San Salvatore" L'Aquila Italy; ^3^ Department of Biotechnological and Applied Clinical Sciences University of L'Aquila L'Aquila Italy

**Keywords:** CD19^+^/CD5^+^ clone, lymphocytic leukemia, SARS‐CoV‐2

## Abstract

Although lymphopenia is currently considered a good predictor for the prognosis of COVID‐19, it must be critically evaluated in patients with CLL, where other clinical markers should be considered to define the prognosis and treatment.

## Graphical abstract

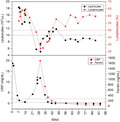

## 1 INTRODUCTION

We report a case of a 64‐year‐old man with B‐cell CLL and other comorbidities who became infected by SARS‐CoV‐2 during hospitalization for spondylodiscitis. Antiviral therapy for this patient was restricted to 1 week due to health concerns, and SARS‐CoV‐2 infection resulted in the transient disappearance of his CD19^+^/CD5^+^ B‐lymphocyte CLL‐characterizing clone for the duration of infection.

In less than 1 year, the coronavirus disease 2019 (COVID‐19) pandemic has caused more than 54 million confirmed cases and over one million deaths.^1^ Individuals with malignancies are considered high‐risk patients for bacterial, fungal, and viral infections. However, to date, few data are available concerning the incidence and outcome of COVID‐19 in patients with chronic lymphocytic leukemia (CLL).^1^, ^2^ In an Italian survey involving 33 hematology centers and a minimum of 100 patients per centers, 47 of 9930 CLL patients (< 0.5%) were positive to COVID‐19.^3^ A multicenter international study including 43 centers and 198 CLL patients with symptomatic COVID‐19 revealed an overall case fatality rate of 33%.^4^

## 2 CASE PRESENTATION

A 64‐year‐old man was admitted to "San Salvatore" Regional Hospital, L'Aquila, Italy, with lumbosacral pain, a body temperature above 38°C, nausea, dysuria, and strangury. A review of the patient's clinical history revealed untreated chronic monoclonal B lymphocytes lymphocytic leukemia (Rai stage II, immunophenotype: CD19^+^, CD5^+^, CD22^+^, CD23^+^, CD103 negative, and FMC7 negative), type II diabetes, hypertension, and vertebral instability, and he was on ACE2 inhibitor ramipril (2.5 mg) therapy and metformin (500 mg twice a day) glycemic control therapy for type II diabetes.

Hematological parameters, at presentation, were consistent with the previous CLL diagnosis (leukocytes 18.33 × 10^3^/µL, comprising 56.5% lymphocytes and 40.1% neutrophils of the total white blood cell count) and biochemical alterations included: glycemia (145 mg/dL), C‐reactive protein (18.73 mg/dL), and an erythrocyte sedimentation rate of 120 mm/h. Estimated glomerular filtration rate, serum electrolyte levels, and cardiac, muscle, pancreatic, and liver enzyme levels were all within physiological limits, except for gamma‐glutamyltransferase (72 UI/L), which was moderately above the upper physiological limit, and coagulative indices and plasma brain natriuretic peptide levels were also within physiological limits. NMR of the lumbosacral rachis was compatible with suspected spondylodiscitis, and chest X‐rays did not demonstrate lung alterations.

**FIGURE 1:**
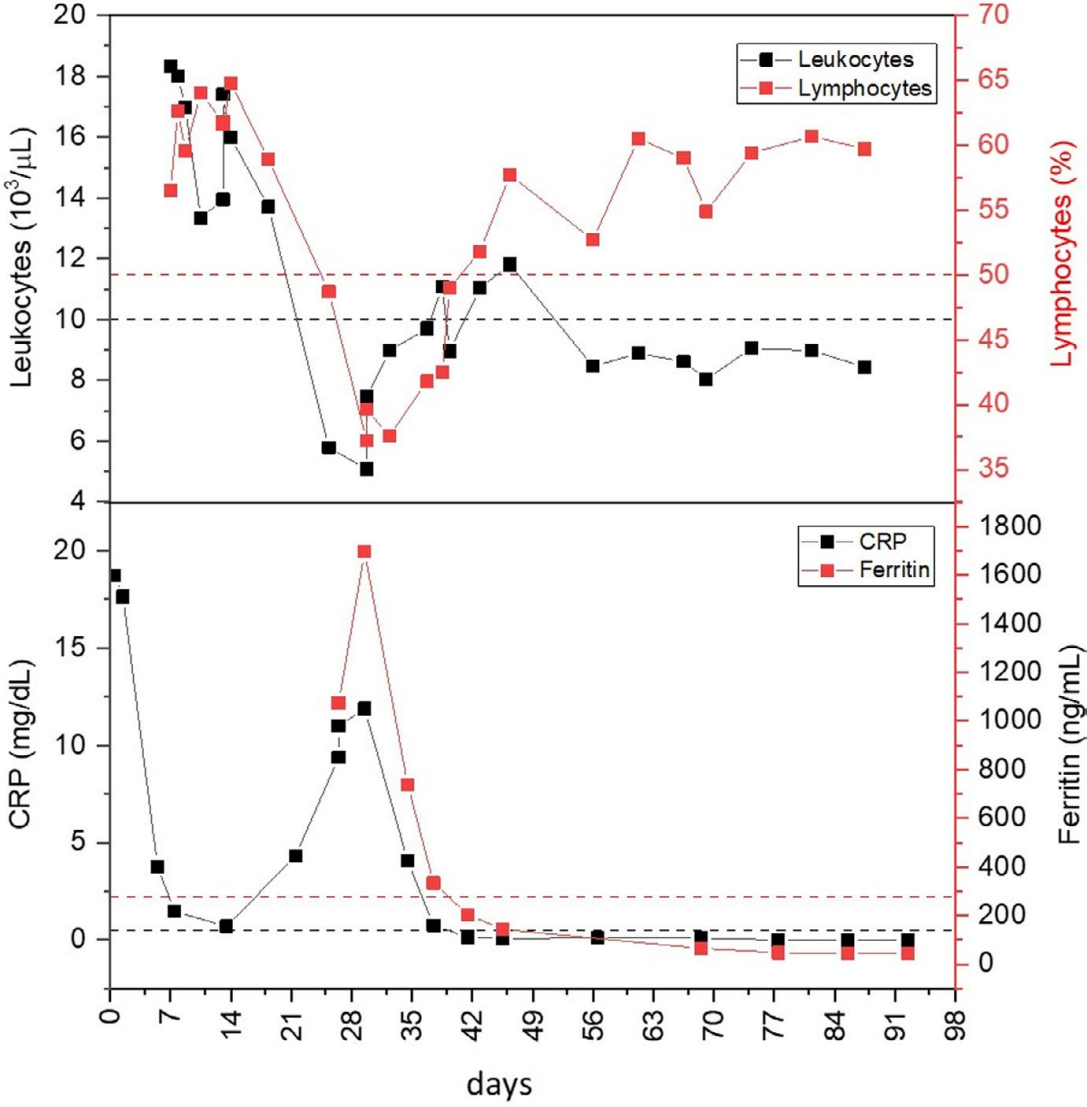
Aligned time‐course plots of significative hematological parameters (leukocytes count and lymphocytes percentage) and inflammation markers (C‐reactive protein and ferritin) recorded from hospital admission. Dashed lines represent the upper limit of the reference ranges

The patient was treated with levofloxacin (750 mg/d) and teicoplanin (400 mg/d) for 4 weeks and oral pregabalin 75 mg/d, oxycodone/naloxone 5/2.5 mg/d, and tramadol/paracetamol 37.5/325 mg/d for pain relief. Thirteen days following admission, the patient's CRP levels had dropped to 0.7 mg/dL and, with unchanged hematological parameters, he was transferred from Internal Medicine to long‐term care. Twenty‐one days following admission, the patient was diagnosed as SARS‐CoV‐2 positive by real‐time RT‐PCR of a nasopharyngeal swab and was moved to the Infectious Diseases department. The patient was asymptomatic but exhibited an increase in CRP levels to 4.32 mg/dL and a decrease in leukocyte count to 5.79 × 10^3^/µL. Five days later (26 days from admission), the patient's arterial oxygen saturation (SaO_2_) levels dropped to ≈90%, chest CT revealed prevalently peripheric bilateral ground‐glass opacities, compatible with new coronavirus infection. Blood CRP and ferritin levels rapidly increased to reach maximums of 11.89 mg/dL and 1695.6 mg/dL, respectively, within 3 days, preceded by a pronounced reduction in leukocytes and lymphocytes numbers, during infection, to within the physiological range (30.6% neutrophils and 64.8%lymphocytes at admission to 58.5% neutrophils and 37.2% lymphocytes during SARS‐CoV‐2 infection; Figure 1).

The patient was successfully treated with darunavir (800 mg/d), ritonavir (100 mg/d), and with hydroxychloroquine (200 mg twice per day) for 1 week, by which time his CRP and ferritin levels had returned to the physiological range, in the absence of other biochemical changes. Three months following infection, the patient was asymptomatic, exhibited biochemical and coagulative indices, and arterial blood gas tests within the physiological range and, despite a marked anti‐SARS‐CoV‐2 IgG humoral immune response, remained positive for SARS‐CoV‐2 for up to 3 months following the disappearance of symptoms. Between the thirty‐fifth and seventy‐first days, following COVID‐19 diagnosis, the patient's mean (±SD) leukocytes count was 8.64 × 10^3^ ± 0.36/µL and mean (±SD) neutrophil count was 3.09 × 10^3^ ± 0.20/µL, associated with a moderate mean (±SD) lymphocytosis of 5.02 × 10^3^ ± 0.46/µL), resulting in a typical differential mean (±SD) WBC of 58.13 ± 3.08% neutrophils and 35.86 ± 3.10% lymphocytes (Figure 1).

## 3 DISCUSSION

In this case study, we report the biochemical, hematological, and coagulative changes in a COVID‐19 patient presenting with stage 2 CLL, diabetes, and hypertension; three conditions reported negatively affect the clinical COVID outcome over the whole duration of SARS‐CoV‐2 infection.

The mortality rate for COVID‐19 depends on several factors and ranges between 2% and 5%.^5^ Fatal outcomes for COVID‐19 associate more frequently with comorbidities and/or immunosuppression, including hematological malignancies.^6^ CLL is more prevalent in the elderly, is characterized by immunodeficiency, and often associates with comorbidities. Although relatively little is known about the clinical course of COVID‐19 in CLL patients, estimates suggest that around 30‐33% of CLL patients with symptomatic SARS‐CoV‐2 infection have a poor outcome and, in general, are considered to be more susceptible to severe disease.^6^, ^7^, ^8^ The deregulation of adaptative immunity in CLL results in humoral immunodeficiency, even in the absence of disease progression, and is likely, therefore, to influence antibody responses.^9^ Hypertension and diabetes represent comorbidities that are more frequently associated with a fatal outcome in COVID‐19, which in Italy have been associated with 73.8% and 33.9% COVID‐19 deaths, respectively (Characteristics of COVID‐19 patients dying in Italy. Report based on available data on March 20th, 2020, National Institute of Health, https://www.epicentro.iss.it/en/coronavirus/sars‐cov‐2‐analysis‐of‐deaths). Notwithstanding these statistics and the complete absence of therapeutic recommendations for COVID‐19 in patients with lymphoid malignancies, which became available only months later,^10^, ^11^ this particular CLL patient who also presented with diabetes and hypertension was efficaciously treated for COVID‐19 in the empirical strategy described above.

Forty‐five days following COVID‐19 diagnosis, the patient exhibited a strong IgG, but not IgM, anti‐SARS‐CoV‐2 humoral immune response, attributable to the low titer at the time of analysis, a transient immune response not captured within the testing window and suboptimal seroconversion in CLL patients.^12^ Nevertheless, this patient remained SARS‐CoV‐2 positive for 3 months following the disappearance of symptoms, suggesting that CLL‐induced immunodeficiency might negatively affect viral clearance, despite the IgG seroconversion.

Surprisingly, the leukocytosis detected at hospital admission, compatible with CLL, reduced dramatically during SARS‐CoV‐2 infection to within the physiological range and remained at this level for up to 3 months, and the CD19^+^/CD5^+^ CLL clone that characterized this patient's disease disappeared, resulting in a physiological neutrophil‐to‐leukocyte ratio and transient remission of CLL. Lymphopenia and neutrophilia characterize COVID‐19 in non‐CLL patients and correlate with COVID‐19 intensive care unit admissions, ARDS and disease severity.^13^, ^14^, ^15^, ^16^ It is, therefore, possible that lymphopenia and neutrophilia induced by SARS‐CoV‐2 infection may explain the transient CLL remission exhibit by the patient in this report. In support of this hypothesis, Vardanyan and colleagues reported a case of a 61‐year‐old CLL patient (Binet stage A), who experienced a partial 6 days resolution of CLL lymphocytosis during SARS‐CoV‐2 infection^17^ and, based on previous studies,^18^ implicated a potential antiproliferative role for IL‐6. Within this context, it is also worth noting that an antitumor immune response resulting from infection‐induced inflammatory cytokine expression has been hypothesized in SARS‐CoV‐2‐induced remission of Hodgkin lymphoma.^19^ It is, therefore, conceivable that the proinflammatory response triggered by SARS‐CoV‐2 infection may have been responsible for the transient disappearance of the CD19^+^/CD5^+^ CLL clone in this patient. This hypothesis is corroborated by the maintenance of white blood cell numbers within the physiological range for the duration of SARS‐CoV‐2 infection in this CLL patient.

## 4 CONCLUSION

Based on the patient's clinical history and comorbidities (CLL, diabetes, and hypertension) associated with poor prognosis in COVID‐19, the patient's optimum outcome in this case report was completely unexpected and cautions against directly generalizing from previous research finding should be taken. The distinctive kinetics and tropism patterns of SARS‐CoV‐2 in patients with underlying hematological malignancies call for specific clinical and diagnostic management strategies. Even though lymphopenia and associated neutrophilia are valuable prognostic markers and useful therapeutic efficacy indices, other biochemical markers must also be carefully considered in COVID‐19 patients with hematological malignancies of the immune system.

## CONFLICT OF INTEREST

None declared.

## AUTHOR CONTRIBUTION

RB, GDM, and AC involved in data collection, GC, PB, and AP involved in data analysis and interpretation. MP and GA critically revised the manuscript. GC wrote the manuscript.

## ETHICAL APPROVAL

This case study does not include any scientific trials involving human subjects.

## Data Availability

The data that support the findings of this study are available from the corresponding author upon request.
